# First antimicrobial resistance data and genetic characteristics of *Neisseria gonorrhoeae* isolates from Estonia, 2009–2013

**DOI:** 10.1002/nmi2.57

**Published:** 2014-08-03

**Authors:** D Golparian, T Brilene, Y Laaring, E Viktorova, E Johansson, M Domeika, M Unemo

**Affiliations:** 1 WHO Collaborating Centre for Gonorrhoea and other Sexually Transmitted Infections, National Reference Laboratory for Pathogenic Neisseria, Department of Laboratory Medicine, Microbiology, Örebro University HospitalÖrebro, Sweden; 2 Department of Microbiology, University of TartuTartu, Estonia; 3 Pärnu HospitalPärnu, Estonia; 4 Central Laboratory of Communicable Diseases, Estonian Health BoardTallinn, Estonia; 5 Department of Prevention and Control of Communicable Diseases, Uppsala County CouncilUppsala, Sweden

**Keywords:** Antimicrobial resistance, Estonia, gonorrhoea, *Neisseria gonorrhoeae*, *N. gonorrhoeae* multiantigen sequence typing

## Abstract

Gonorrhoea is a sexually transmitted infection with major public health implications and *Neisseria gonorrhoeae* has developed resistance to all antimicrobials introduced for treatment. Enhanced surveillance of antimicrobial resistance in *N. gonorrhoeae* is crucial globally. This is the first internationally reported antimicrobial resistance data for *N. gonorrhoeae* from Estonia (44 isolates cultured in 2009–2013). A high prevalence of resistance was observed for azithromycin, ciprofloxacin and tetracycline. One and two isolates with resistance and decreased susceptibility to the last remaining first-line treatment option ceftriaxone, respectively, were identified. It is crucial to implement surveillance of gonococcal antimicrobial resistance (ideally also treatment failures) in Estonia.

Gonorrhoea is a sexually transmitted infection with major public health implications. The WHO estimated that 106 million new cases of gonorrhoea occurred among adults worldwide in 2008, which represented a 21% increase compared with 2005 1. Gonorrhoea, including its severe complications, results in substantial morbidity and economic costs globally. In Estonia, the reported annual incidence (cases per 100 000 population) of gonorrhoea showed an all-time high in 1993 (233.9). The incidence subsequently decreased annually to 8.8 in 2010; although there was a slight increase to 12.4 in 2011, which was the fourth highest incidence in the European Union in 2011 2. The first-line empiric treatment of gonorrhoea in Estonia is ceftriaxone (250 mg intramuscularly) 3; however, in practice a variety of antimicrobials can be used (particularly among private practitioners), e.g. cefixime, azithromycin, fluoroquinolones or tetracyclines. The emergence of treatment failures to the last remaining option for treatment, i.e. ceftriaxone, 4–9 and extensively drug resistant *Neisseria gonorrhoeae* strains 9–11 has placed antimicrobial resistance (AMR) surveillance as an essential key priority nationally and internationally; however, no AMR data for *N. gonorrhoeae* has ever been internationally reported from Estonia.

In this study, we investigated the AMR to previously and currently recommended treatment options and genotypic characteristics of *N. gonorrhoeae* isolates in 2009–2013 in Estonia.

Forty-four clinical *N. gonorrhoeae* isolates were tested from 2009 (*n* = 5), 2010 (*n* = 6), 2011 (*n* = 5), 2012 (*n* = 15) and 2013 (*n* = 13). The isolates were cultured from mainly symptomatic gonorrhoea patients (17 women, 26 men and 1 unknown) attending different dermatovenereological clinics in Estonia. Mean age for the women was 25 years (median 25 years; range 16–40 years) and for the men 28 years (median 29 years; range 22–40 years). Twenty-five (56.8%) isolates were obtained from specimens from male urethra, 17 (38.6%) from cervix, one (2.3%) from male rectum, and one unknown (2.3%). All isolates were cultured, species was verified and samples were preserved as previously described 12. The MICs (mg/L) of ceftriaxone, cefixime, azithromycin, spectinomycin, ciprofloxacin, tetracycline and gentamicin were analysed using the Etest method, according to the instructions from the manufacturer (BioMérieux AB, Solna, Sweden). All results were interpreted using whole MIC dilutions and breakpoints for susceptibility (S) and resistance (R) according to the European Committee on Antimicrobial Susceptibility Testing (EUCAST: www.eucast.org). Furthermore, isolates with a cephalosporin MIC of >0.064 to 0.125 mg/L have resulted in treatment failures 4–8 and, accordingly, these isolates were considered to have a decreased susceptibility to cefixime and ceftriaxone. The *penA* gene, encoding the lethal cephalosporin target penicillin-binding protein 2, was sequenced as previously described 13. For gentamicin, no breakpoints are stated by any organization. *β*-lactamase production was identified using nitrocefin solution. Bacterial DNA was isolated using the NorDiag Bullet instrument with the BUGS'n BEADS™ STI-*fast* kit (NorDiag ASA Company, Oslo, Norway), according to the instructions from the manufacturer. Genotyping by *N. gonorrhoeae* multiantigen sequence typing (NG-MAST) was performed as previously described 13,14. The *penA* gene, the main cephalosporin-resistance determinant, was sequenced in isolates displaying decreased susceptibility or resistance to ceftriaxone and categorized as previously described 9,13. The 2008 WHO *N. gonorrhoeae* reference strains 13 were used for quality control in all phenotypic and molecular characterization.

The results of the AMR testing of all isolates are summarized in Table1. Briefly, the overall proportions of isolates with resistance were as follows: ceftriaxone 2.3%, azithromycin 22.7%, ciprofloxacin 27.3% and tetracycline 34.1%. No isolates resistant to cefixime or spectinomycin were identified, and the MICs of gentamicin were low (MIC range 2–8 mg/L). Eight (18.2%) of the isolates showed multidrug resistance, i.e. resistance to azithromycin, ciprofloxacin and tetracycline. Seven (15.9%) of the isolates were *β*-lactamase producing. One (2.3%) isolate with resistance to ceftriaxone (MIC = 0.25 mg/L) and two (4.5%) isolates displaying decreased susceptibility to ceftriaxone (MIC = 0.125 mg/L 12,15) were identified. All these three isolates possessed a *penA* mosaic allele XXXIV 9, explaining the enhanced MICs of ceftriaxone. Furthermore, two of these isolates had a decreased susceptibility also to cefixime (MIC = 0.125 mg/L), while the third isolate was susceptible to cefixime (MIC = 0.032 mg/L). No additional isolates displayed a decreased susceptibility to cefixime. Among the 44 isolates, 18 different NG-MAST sequence types (STs) were identified, of which nine (50%) STs have not been previously described (Table2). Nine (50%) of the STs were represented by more than one isolate. ST1241 was the most prevalent genotype, and all ST1241 isolates (*n* = 14) were susceptible to all tested antimicrobials. Isolates of the second most prevalent genotype, ST2212 (*n* = 6), were resistant to azithromycin, ciprofloxacin and tetracycline, and one of these ST2212 isolates also showed a decreased susceptibility (MIC = 0.125 mg/L) to ceftriaxone and cefixime (Table2).

**Table 1:** Antimicrobial susceptibility of 44 *Neisseria gonorrhoeae* isolates from Estonia, 2009–2013

Antimicrobial breakpoints (mg/L)^a^	S (%)	I (%)	R (%)
Ceftriaxone (S ≤ 0.125, R >0.125)	43 (97.7)	NA	1 (2.3)
Cefixime (S ≤0.125, R >0.125)	44 (100)	NA	0
Spectinomycin (S ≤64, R >64)	44 (100)	NA	0
Azithromycin (S ≤0.25, R >0.5)	24 (54.5)	10 (22.7)	10 (22.7)
Ciprofloxacin (S ≤0.032, R >0.064)	32 (72.7)	0	12 (27.3)
Tetracycline (S ≤0.5, R >1.0)	27 (61.4)	2 (4.5)	15 (34.1)
Gentamicin^b^	MIC range: 2–8 mg/L; MIC_50_: 4 mg/L; and MIC_90_: 4 mg/L		
β-lactamase production	2009: 0 (0%); 2010: 1 (16.7%); 2011: 2 (40.0%); 2012: 4 (26.7%); and 2013: 0 (0%)		

S, susceptible; I, intermediate susceptible; R, resistant; NA, not applicable.

a Breakpoints according to The European Committee on Antimicrobial Susceptibility Testing (EUCAST; www.eucast.org).

b Breakpoints not stated by any organization.

**Table 2:** Molecular epidemiological characteristics of *Neisseria gonorrhoeae* isolates in Estonia, 2009–2013

NG-MAST	Year					Total
	2009	2010	2011	2012	2013	
ST211				2		2^a^
ST437		1				1^b^
ST1241	2	1	1	5	5	14
ST2212	1	1			4^b^	6^c^
ST2449				1		1^d^
ST3611			2			2^a^
ST4120				1		1^b^
ST5185				1		1
ST7482^e^			2			2
ST7483^e^	2					2
ST7484^e^		1				1^d^
ST7485^e^		1		1		2
ST7486^e^				1		1
ST7487^e^		1^f^		2^f^		3^g^
ST7488^e^				1		1
ST8392					1	1^f^
ST9900^a^					2	2
ST9901^a^					1	1
Total	5	6	5	15	13	44

NG-MAST, *Neisseria gonorrhoeae* multiantigen sequence type; ST, sequence type.

a All isolates resistant to ciprofloxacin and tetracycline, and producing β-lactamase.

b Including isolates with decreased susceptibility (MIC = 0.125 mg/L 11,18) or resistance (MIC >0.125 mg/L) to extended-spectrum cephalosporins, including resistance to azithromycin (MIC >0.5 mg/L), ciprofloxacin (MIC >0.064 mg/L) and tetracycline (MIC >1 mg/L).

c All isolates resistant to azithromycin, ciprofloxacin and tetracycline.

d Isolates resistant to azithromycin.

e Not previously described sequence types.

f Isolates resistant to tetracycline.

g All isolates producing *β*-lactamase.

In the present study, the susceptibility to previously and currently recommended antimicrobials for treatment of gonorrhoea was investigated in *N. gonorrhoeae* isolated in 2009–2013 in Estonia. Despite the global concern over extensively drug resistant gonorrhoea and the possibility of untreatable gonorrhoea in the future 9, this is the first internationally reported AMR data for *N. gonorrhoeae* from Estonia. A high prevalence of resistance was observed for azithromycin (22.7%), ciprofloxacin (27.3%) and tetracycline (34.1%), but no resistance to cefixime or spectinomycin was found (Table1). Worryingly, one (2.3%) and two (4.5%) isolates with resistance and decreased susceptibility, respectively, to the last remaining first-line treatment option ceftriaxone were identified. All these three isolates possessed the *penA* mosaic allele XXXIV, which has been associated with ST1407 or closely related STs (genogroup 1407 15–19) that have accounted for most decreased susceptibility or resistance to extended-spectrum cephalosporins (ESCs) in many European countries and mainly globally, and resulted in most verified ESC treatment failures 4,10–12,17,16,18,15,19. Moreover, two of these isolates, assigned as ST2212 and ST4120, also belonged to this genogroup 1407, which additionally is multidrug resistant 19. The remaining isolate, assigned as ST437, has also been previously associated with decreased ESC susceptibility 17,20,21,18,15,19. The most prevalent ST (*n* = 14) in this study, ST1241, has been previously described in Italy in *N. gonorrhoeae* isolated from heterosexual males in 2003–2005 17. However, in that study the ST1241 isolates displayed resistance to ciprofloxacin and tetracycline, whereas in this study all the isolates (*n* = 14) were susceptible to both these antimicrobials. This highlights the caution required when using genotyping, such as NG-MAST, only for prediction and surveillance of AMR.

In conclusion, it is crucial to continuously follow the spread of gonococcal strains with multidrug resistance and decreased susceptibility or resistance to ESCs in Estonia, and implement quality-assured culture-based surveillance of gonococcal AMR (ideally also treatment failures) in Estonia.
